# The role of *pmrCAB* genes in colistin-resistant *Acinetobacter baumannii*

**DOI:** 10.1038/s41598-022-25226-x

**Published:** 2022-12-05

**Authors:** Shaimaa Mohamed Seleim, Marwa Salah Mostafa, Nadia Hafez Ouda, Rania Yahia Shash

**Affiliations:** Department of Medical Microbiology and Immunology, Faculty of Medicine, Cairo University, Cairo, Egypt

**Keywords:** Microbiology, Molecular biology, Health care, Medical research

## Abstract

The progressively increasing antimicrobial-resistant *Acinetobacter baumannii* infections have enforced the use of colistin as the last option for therapy, resulting in the colistin resistance evolution. This work aimed to study the *pmrCAB* expression in *A. baumannii* isolates as well as the presence of the *mcr-1* gene. Colistin MICs of 100 *A. baumannii* isolates were measured using the broth microdilution assay. In four colistin-susceptible and four colistin-resistant isolates, the relative expression of the *pmrA, pmrB*, and *pmrC* genes was determined using reverse transcription PCR, and then selected isolates were sequenced using the Sanger technique. Finally, the *mcr-1* gene was detected using conventional PCR. The colistin resistance rate among the studied isolates was 49%. The expression levels of *pmrA* and *pmrB* were statistically significantly higher in colistin-resistant isolates than in colistin-susceptible ones, while the *pmrC* expression had no statistically significant change. There was a weak positive correlation between colistin MICs and the expression levels of each of the *pmrA* and *pmrB* genes. By sequencing, two colistin-resistant strains with low *pmrCAB* expression showed insertion mutations 3277188_3277189T in *pmrB* and 1185149_1185150T in *pmrC*. Only one isolate (1%) was positive for the presence of *mcr-1*. We concluded that *pmrCAB* increased expression and/or mutations may cause colistin resistance in *A. baumannii*. However, increased *pmrC* expression may not necessarily result in colistin resistance. In Egypt, this is the first study to reveal the existence of *mcr-1* in *A. baumanni*. This should attract attention in clinical settings due to the ultimate tendency of spreading colistin resistance.

## Introduction

*Acinetobacter baumannii* causes serious hospital-acquired infections like respiratory tract, urinary tract, bloodstream, surgical site, and wound infections. The abuse of antibiotics has led to the rise of extensively drug-resistant strains, forcing clinicians to utilize colistin as the last treatment option to fight these infections^1,2^.

Colistin is cyclic, positively charged peptide antibiotic with bactericidal activity against several Gram-negative bacteria due to its interaction with the negatively charged lipid A part of lipopolysaccharide (LPS). So, the bacterial outer membrane (OM) integrity is affected, resulting in cytoplasmic fluid leakage and cell death^3^.

Unfortunately, the surge in colistin usage in treating infections caused by multi-drug resistant *A. baumannii* resulted in colistin resistance spread^4^. The most important colistin resistance mechanisms identified in *A. baumannii* are (1) the LPS modification mediated by the two-component system PmrA/PmrB and (2) the LPS loss caused by lipid A impaired synthesis^5,6^. Recently, a plasmid-mediated colistin resistance gene has been also involved in colistin resistance^7^. The *pmrA*/*pmrB* encodes a two-component response regulator and sensor kinase system that helps bacteria to sense and respond to environmental factors. In addition, they influence the *pmrC* gene expression, which encodes lipid A phosphoethanolamine (PEtN) transferase that adds PEtN to the lipid A part of the LPS. LPS modification causes less negative charge and decreased colistin binding affinity to its target on the LPS^5,8^.

The *mcr-1* is a plasmid-mediated gene that encodes a PEtN transferase that causes colistin resistance^9^. In 2015, it was found in *Escherichia coli* obtained from a variety of clinical, animal, and environmental specimens from China^10^. Furthermore, *mcr-1* was discovered for the first time in *P. aeruginosa* and *A. baumannii* isolated from a variety of clinical samples in Pakistan^7^.

The aim of the present study was to investigate the expression of *pmrCAB* and mutations in *A. baumannii* isolates, as well as the existence of the plasmid-encoded *mcr-1*.

## Materials and methods

We conducted a descriptive cross-sectional study from January 2020 to March 2021. The Clinical Laboratories of Cairo University hospitals in Cairo, Egypt provided 100 *Acinetobacter* species isolates. They were obtained from different clinical specimens, including purulent discharge, sputum, pleural fluid, urine, blood, and ascitic fluid. All isolates were transferred to the Medical Microbiology and Immunology Department, Faculty of Medicine, Cairo University for confirmation and further processing. The research was authorized by the Ethics Committee of the Institutional Review Board (Code: MD-254–2019), Faculty of Medicine, Cairo University, Egypt.

I. **Culture and identification of *Acinetobacter* isolates**
  Each of the collected isolates was sub-cultured on MacConkey’s medium and then incubated aerobically at 37 °C for 24 h. Identification of *Acinetobacte*r spp. was done by conventional biochemical reactions. *Acinetobacter* was identified as colourless or light lavender colonies, non-motile, Gram-negative, catalase-negative, oxidase-negative, non-fermentative cocco-bacilli^11^.
II. **Molecular identification of *Acinetobacter baumannii***
  Following the manufacturer's instructions, DNA extraction from all *Acinetobacter* isolates was done using the GF-1 Bacterial DNA Extraction Kit (Vivantis, Malaysia). Using PCR, amplification of the *A. baumannii* species-specific *bla*_*OXA-*51-like_ gene was done^12^. It was performed using a ready-to-use PCR master mix (CinnaGen, Iran). Specific primers for the *bla*_*OXA-*51-like_ gene (forward primer: TAATGCTTTGATCGGCCTTG, and reverse primer: TGGATTGCACTTCATCTTGG) were used^12^. The DNA amplification was performed in Rotor-Gene Q MDX (Qiagen, Germany). The cycling conditions were as follows: initial denaturation at 94 °C for 3 min, followed by 34 cycles of denaturation at 94 °C for 30 s, primer annealing at 55 °C for 30 s, and extension at 72 °C for 30 s, followed by a final extension at 72 °C for 5 min^12^. The amplified PCR products (353 bp) were run in agarose gel electrophoresis along with a 50 bp molecular weight ladder (Vivantis, Malaysia) and then seen under an ultra-violet (UV) transilluminator.
III. **Detection of colistin resistance**
  According to the European Committee on Antimicrobial Susceptibility Testing (EUCAST) guidelines, the colistin minimal inhibitory concentration (MIC) of each *A. baumannii* isolate was evaluated using the broth microdilution method with Cation-Adjusted Muller Hinton Broth (CA-MHB) (Iiofilchem, Italy)^13^. Using EUCAST resistance breakpoints for *A. baumannii*, colistin resistance was considered if MIC is > 2 mg/L^13^.
IV. **Detection of the expression of *pmrA*, *pmrB* and *pmrC* genes**
  The relative expression of *pmrA*, *pmrB* and *pmrC* genes was measured using qRT-PCR. RNA extraction was done using HiPurA™ Bacterial RNA Purification Kit (HiMedia, India) according to the manufacturer’s instructions. The extracted RNA was used for the synthesis of cDNA using SinaClon First Strand cDNA synthesis Kit (CinnaGen, Iran).
  The expression levels of *pmrA*, *pmrB,* and *pmrC* genes were measured using the primer sets shown in Table 1^14^. Quantitative RT-PCR was done using Thermo Scientific Maxima SYBR Green/ROX qPCR Master Mix (2X) (Thermofisher Scientific, US).
  The prepared reaction mixtures were processed in Rotor-Gene Q MDX with software version 2.3.3 (Qiagen, Germany) for amplification and analysis. After the qRT-PCR run, data were expressed in Cycle threshold (Ct). The target genes’ expression levels were normalized using the housekeeping 16S rRNA gene expression then their analysis was finalized using the comparative 2^−∆∆CT^ process.
V. **DNA sequencing of *pmrA*, *pmrB,* and *pmrC* genes**
  Eight *A. baumannii* isolates were selected for DNA sequencing. These isolates included two colistin-resistant isolates with high *pmrCAB* expression (isolates #16 and 41), two colistin-resistant isolates with low *pmrCAB* expression (isolates # 85 and 71), two colistin-susceptible isolates with high *pmrCAB* expression (isolates # 1 and 44) and two colistin-susceptible isolates with low *pmrCAB* expression (isolates # 4 and 43).
  A portion of each of *pmrA*, *pmrB,* and *pmrC* genes was amplified using the primers shown in Table 1^15^. The amplified PCR products were sent to Color Laboratory, Egypt, for Sanger sequencing. BigDye® XTerminator™ Purification Kit (Applied Biosystems, USA) was used for purifying the amplified PCR products according to the instructions of the manufacturer. Cycle DNA sequencing was done in the forward direction using BigDye™ Terminator v3.1 Cycle Sequencing Kit (Thermo Scientific, USA) according to the manufacturer’s instructions. The sequenced products were run on a 3500 Genetic Analyzer (Applied Biosystems, USA).
VI. **Detection of the plasmid-encoded *mcr-1***
  All *A. baumanni* isolates were subjected to PCR to amplify the *mcr-1* gene using the following primers: the forward primer: CGGTCAGTCCGTTTGTTC, and the reverse primer: CTTGGTCGGTCTGTAGGG^5,7^. The DNA amplification was performed in Rotor-Gene Q MDX (Qiagen, Germany)***.*** The amplified product (309 bp) was run in agarose gel electrophoresis along with a 50 bp molecular weight ladder (Cleaver Scientific, UK) and then seen under an ultra-violet (UV) transilluminator. The positive control used in this study was an *Escherichia coli* isolate carrying the *mcr-1* gene previously identified by sequencing. It was supplied by the Medical Microbiology and Immunology Department, Faculty of Medicine, Cairo University.

**Table 1:** Primer sequences used for the amplification of *pmrA*, *pmrB,* and *pmrC.*

Target genes	Primer	Sequence (5′–3′)	Amplicon size (bp)
*pmrA*	Forward	ATGACAAAAATCTTGATGATTGAAGAT	175
	Reverse	CCATCATAGGCAATCCTAAATCCA	
*pmrB*	Forward	GAACAGCTGAGCACCCTTTAA	145
	Reverse	ACAGGTGGAACCAGCAAATG	
*pmrC*	Forward	CTCTTTACGCTTTGTTTTATGGAC	159
	Reverse	GTAAAAAGTAAAACACCGACCA	
*16S rRNA*	Forward	TCAGCTCGTGTCGTGAGATG	
	Reverse	CGTAAGGGCCATGATG	

### Statistical analysis

Data were analyzed using the statistical package for the Social Sciences (SPSS) version 26 (IBM Corp., Armonk, NY, USA). Quantitative data were summarized using the mean and standard error of the mean (SEM), whereas categorical data were summarized using frequency (count) and relative frequency (percentage). For comparing quantitative variables, the non-parametric Mann–Whitney test was utilized. For comparing categorical data, the Chi-square (2) test was utilized. When the anticipated frequency was < 5, the exact test was used instead.

To calculate correlations between quantitative variables, the Spearman correlation coefficient was used. A receiver operating characteristic (ROC) curve was constructed with the area under the curve (AUC) analysis to detect the best cutoff value of RQ of *pmrA*, *pmrB* and *pmrC* genes for discrimination between sensitive and resistant isolates. To determine statistical significance, a probability value (P) equal to or < 0.05 was employed.

### Ethics approval

This study was approved by the Research Ethics Committee of the Institutional Review Board (Code: MD-254–2019), Faculty of Medicine, Cairo University.

## Results

One hundred *Acinetobacter* isolates were obtained from the laboratories of Cairo University hospitals. 65% of the isolates were from patients in ICUs. The isolates were from 48 male and 52 female patients with a mean age of 50.03 ± 20.72 years. Those isolates were collected from various clinical samples including purulent discharge (32), sputum (29), blood (20), urine (14), pleural fluid (3), and ascitic fluid (2). Molecular detection of the *bla*_*OXA-*51-like_ gene confirmed that all isolates (100%) were *A. baumannii.* 49% of the studied *A. baumannii* isolates were colistin-resistant, while 51% were colistin-susceptible (Supplementary Table S1),

### Expression of *pmrCAB* genes

**Figure 1:**
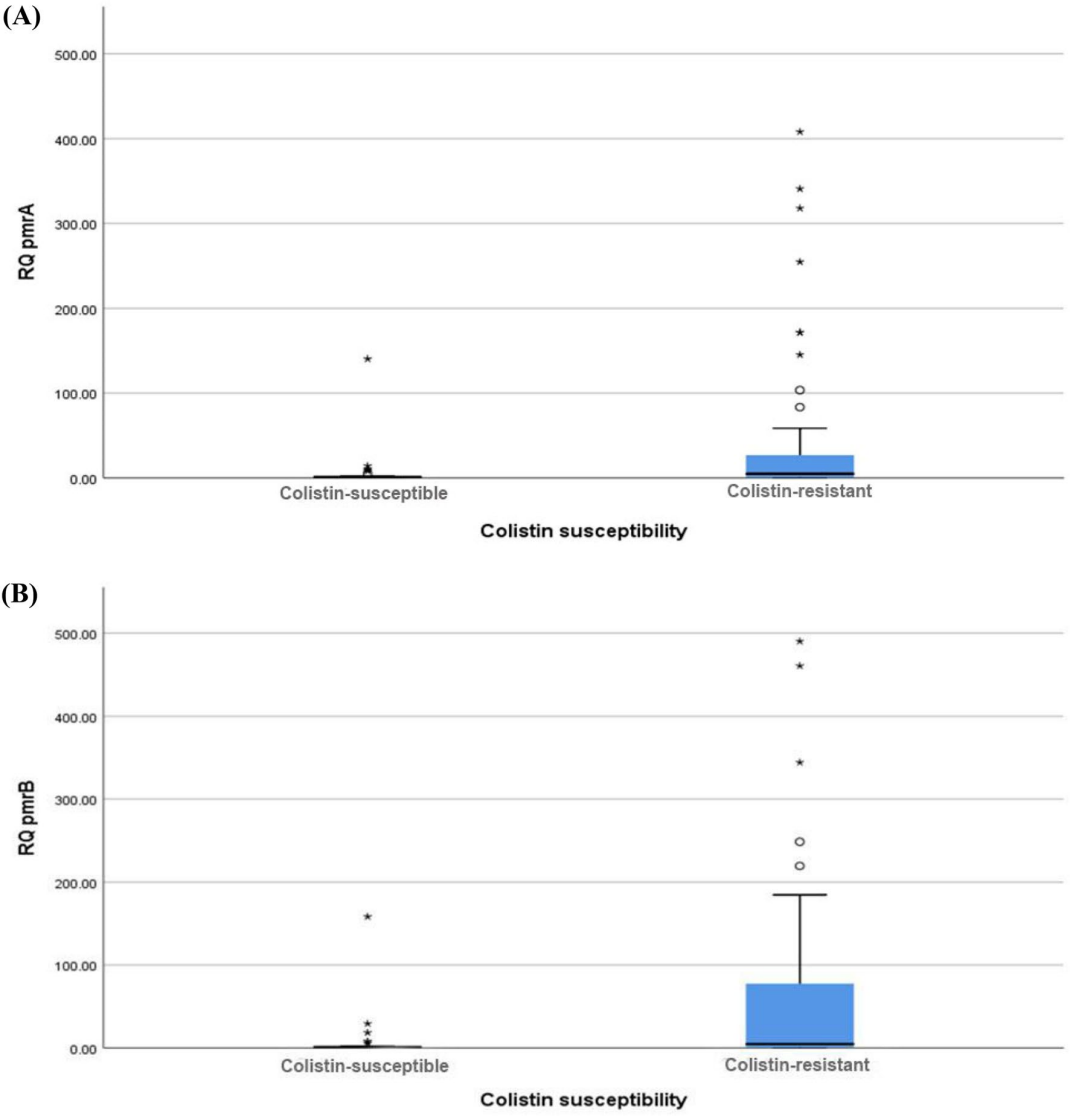
(**A**) Comparison between *pmrA* expression levels in colistin-susceptible and colistin-resistant isolates (P < 0.001). (**B**) Comparison between *pmrB* expression levels in colistin-susceptible and colistin-resistant isolates (P = 0.001).

Analysis of *pmrCAB* expression showed that *pmrA* expression levels were statistically significantly higher in colistin-resistant isolates (mean ± SEM = 46.84 ± 13.78) compared to the colistin-susceptible isolates (mean ± SEM = 4.56 ± 2.74) (*P* < 0.001) (Table 2 and Fig. 1A).

**Table 2:** Expression levels of *pmrA*, *pmrB*, and *pmrC* in colistin-susceptible and colistin-resistant isolates.

	Colistin susceptibility				*P* value
	Colistin sensitive		Colistin resistant		
	Mean	SEM	Mean	SEM	
RQ of *pmrA*	4.56	2.74	46.84	13.78	< 0.001
RQ of *pmrB*	5.25	3.14	64.31	16.52	0.001
RQ of *pmrC*	19.63	16.55	26.69	12.32	0.448

*RQ* relative quantification, *SEM* standard error of mean.

Similarly, *pmrB* expression levels were statistically significantly higher in colistin-resistant isolates (mean ± SEM = 64.31 ± 16.52) compared to the colistin-susceptible isolates (mean ± SEM = 5.25 ± 3.14) (*P* = 0.001) (Table 2 and Fig. 1B). Concerning *pmrC* expression levels, no significant difference was found between colistin-resistant and colistin-susceptible isolates (Table 2). Expression levels of *pmrA*, *pmrB*, and *pmrC* are shown in Supplementary Table S1.

The Spearman correlation coefficient test revealed a weak positive correlation between the MIC values of colistin and the expression levels of each of *pmrA* (r = 0.399, *P* < 0.001) and *pmrB* genes (r = 0.383, *P* < 0.001). However, there was insignificant correlation between *pmrC* expression levels and colistin MICs (r = 0.149, *P* = 0.139).

**Figure 2:**
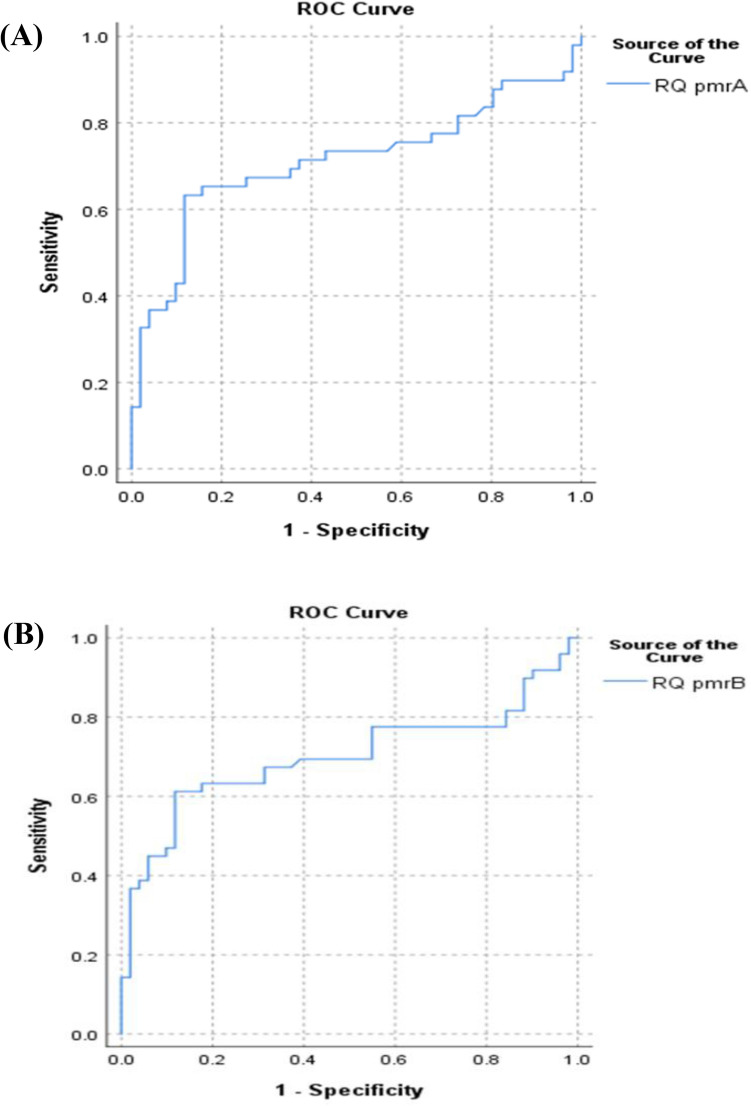
(**A**) Receiver operating characteristic curve of *pmrA* expression (AUC 0.713, *P* < 0.001). (**B**) Receiver operating characteristic curve of *pmrB* expression (AUC = 0.699, *P* < 0.001).

ROC curve analysis revealed that *pmrA* expression could differentiate between colistin-susceptible and colistin-resistant isolates showing sensitivity of 63.3% and a specificity of 88.2% at a cutoff value = 2.7105 (AUC 0.713, *P* < 0.001) (Fig. 2A).

Similarly, the expression levels of *pmrB* could differentiate between colistin-susceptible and colistin-resistant isolates showing sensitivity of 61.2% and a specificity of 88.2% at a cutoff value = 2.3219 (AUC = 0.699, *P* < 0.001) (Fig. 2B). On the other hand, the *pmrC* expression levels could not differentiate between colistin-susceptible and colistin-resistant isolates (AUC = 0.544, *P* = 0.459).

### Sequencing of *pmrCAB* genes

The sequences of the *pmrA, pmrB,* and *pmrC* genes of the 8 isolates were compared with the reference sequence of *A. baumannii* ATCC 19,606 (GenBank: CP045110.1) (https://www.ncbi.nlm.nih.gov/nuccore/CP045110.1).

Insertion mutations were observed in both colistin-resistant isolates with low *pmrC* expression. Isolate number 71 showed 3277188_3277189T in *pmrB.* Isolate number 85 showed 1185149_1185150T in *pmrC*, in addition to two substitutions (1185196 T > G and 1185199A > C).

### Detection of plasmid-mediated *mcr-1* gene

**Figure 3:**
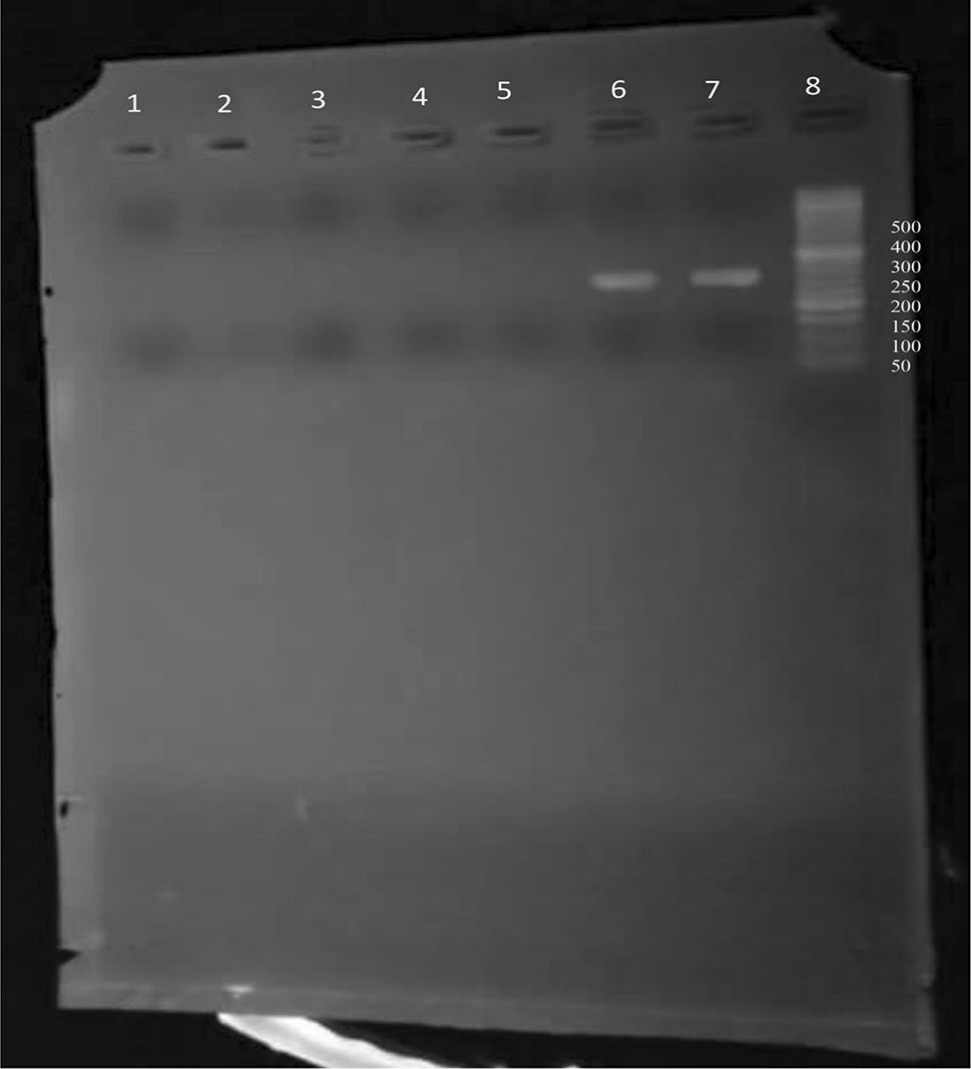
Gel electrophoresis showing the amplified product of the *mcr-1* gene (309 bp). Lanes 1–4: negative isolates; Lane 5: negative control; Lane 6: positive control (band size 309 bp); Lane 7: *mcr-1* positive isolate; Lane 8: 50 bp DNA ladder (50 bp-1500 bp) (Cleaver Scientific, UK).

We found only one (1%) *A. baumannii* isolate that carried *mcr-1* (Fig. 3). The colistin MIC of this isolate was 4 mg/L (Supplementary Table S1).

## Discussion

*A. baumannii* drug resistance is spreading rapidly around the world and is impacted by various factors; one of them is antibiotic misuse and overuse^16^. The increasing development of carbapenem-resistant *A. baumannii* has made colistin one of the last therapeutic options used in treating various infections. Colistin-resistant *A. baumannii* strains have been reported since the reintroduction of colistin in human clinical practice^17^.

Our study showed that 49% *A. baumannii* isolates were colistin-resistant. Similar results have been found by Papathanakos et al.^17^ in Greece (41%) and Gerson et al.^18^ in Germany (48%), while a higher rate was reported by Al-Kadmy et al.^19^ in Iraq (76%). Lower rates were reported by Hameed et al.^7^ in Pakistan (9.6%) and Lukovic et al.^20^ in Serbia (4.3%). The high prevalence of resistance to colistin in the present work could be explained by the fact that our isolates were obtained from hospitalized patients since resistance patterns are more frequent in hospitalized patients due to the overuse of antibiotics as well as the presence of co-morbidities.

In the current study, *pmrA* and *pmrB* gene*s* were statistically significantly overexpressed in colistin-resistant isolates compared to colistin-susceptible isolates. Similar results were reported by previous studies^21–23^. Nevertheless, Sepahvand et al.^15^ although found higher expression of *pmrA* in colistin-resistant isolates than colistin-sensitive isolates, they reported that the expression of *pmrB* had no significant change. In fact, overexpression of the *pmrA* and *pmrB* genes decreases lipopolysaccharide, leading to reduced permeability and colistin ineffectiveness on the *A. baumannii* membrane^15^. Additionally, Colistin resistance in *A. baumannii* is caused by mutations in the *pmrAB* genes^21,24^.

Alternatively, the present study showed that the *pmrC* expression level had no statistically significant change between colistin-susceptible and colistin-resistant isolates. Even though overexpression of *pmrC* is associated with reduction of colistin susceptibility and is frequently caused by mutations in the regulators *pmrAB*^25,26^, increased *pmrC* expression has been reported without association with increased colistin MICs^18^.

Moreover, we noticed a weak positive correlation between colistin MIC values and the expression levels of each of the *pmrA* and *pmrB* genes. However, there was a non-significant correlation between *pmrC* expression levels and colistin MICs. On the contrary, Nurtop et al.^5^ found a positive correlation between *pmrC* expression levels and colistin MIC values.

Adams et al.^25^ demonstrated increased *pmrA* expression in colistin-resistant mutants, which autoregulates the *pmrCAB* promoter. They also reported that partial deletion of *pmrB* in colistin-resistant mutant leads to reversion to a colistin-susceptible phenotype, emphasizing the role of the PmrAB system in the regulation of colistin resistance in *A. baumannii*^25^.

A paucity of studies investigated the *pmrA* and *pmrB* expression in colistin-resistant *A. baumanni*. However, previous research demonstrated that *pmrA* and *pmrB* mutations lead to increased *pmrC* expression and hence the addition of PEtN to lipid A. This observation was detected in both colistin-resistant and colistin-susceptible isolates, suggesting that this resistance mechanism may be strain-specific and that other unknown factors may be involved in resistance to colistin^27^. Furthermore, Nurtop et al.^5^ reported that the same clone isolates have different patterns of expression of *pmrC*, *pmrA,* and *pmrB*. This finding confirms the importance of environmental and host factors on the behavior of gene expression. Colistin use was shown in several studies to be a substantial risk factor for the emergence of chromosomal colistin resistance^28^.

Colistin susceptibility testing is performed by MIC determination, which is the gold standard phenotypic method, but it takes about 24 h to get the results. Molecular methods are often used in the detection of antimicrobial resistance besides phenotypic methods. Molecular methods are expected to replace phenotypic methods in many laboratories because of their higher speed and precision in identifying genetic mechanism(s) for antimicrobial resistance^29^. In the current study, ROC curve analysis revealed that *pmrA* and *pmrB* expressions could differentiate between colistin-susceptible and colistin-resistant isolates, with moderate sensitivity (63.3%, 61.2%) and a considerable specificity (88.2%, 88.2%) at a cutoff value of 2.7105 and 2.3219 for *pmrA* and *pmrB* respectively. On the other hand, the expression levels of *pmrC* could not differentiate between them. To our knowledge, this study is the first to assess the expression of *pmrA*, *pmrB,* and *pmrC* in the detection of colistin resistance in *A. baumannii*. Further studies about molecular detection of colistin resistance by detection of the expression of *pmrA* and *pmrB* are needed.

In the current study, we observed two nucleotide insertions that caused frameshift mutations in *pmrB* and in *pmrC* in the two colistin-resistant isolates with low *pmrC* expression. One of the two isolates also showed two substitutions in *pmrC*. On the other hand, previous studies reported *pmrCAB* mutations in association with increased *pmrC* expression, some of these mutations occurred during treatment with colistin^5,8,18,30^. The two-component *pmrA/pmrB* system regulates the *pmrC* expression and subsequently, mutations of *pmrAB* may increase the *pmrC* expression^3^.

The insertion and substitution mutations detected in this study in the two colistin-resistant isolates with decreased *pmrC* expression indicate that these *pmrB/pmrC* mutations may induce colistin resistance in *A. baumannii* without an increase in gene expression. It is unclear how amino acid mutations in the pEtN transferase influence colistin MICs, but an increase in enzyme function or activity is postulated^18^*.* Nevertheless, other colistin resistance mechanisms cannot be excluded.

The additive effect of mutations suggested that mutation accumulation could be a mechanism for increased antibiotic resistance^27,31^, even in the absence of increased gene expression. It can be clarified by the fact that some mutations may increase the gene function, or the activity of a given gene product (*gain-of-function* mutation) rather than loss of gene function^32,33^.

The *mcr-1* gene, a plasmid-mediated colistin-resistant gene, was discovered in an *E. coli* isolate of animal origin in China. It was then detected nearly worldwide in bacterial isolates from animals^34^, humans^35^, and environmental sources^19^. Resistance transmitted by plasmids has two drawbacks. First, plasmids can bear resistance to several antibiotics. Second, plasmids can spread resistance among the bacteria at a greater rate than that which occurs via spontaneous mutation. Therefore, colistin-resistant bacteria may rapidly become widespread in the clinical setting in the lack of new antibiotics against MDR bacteria^36^.

In the current study, out of the 100 *A. baumannii* isolates only one isolate carried *mcr-1. *Hameed et al.^7^ reported a similar result in Pakistan and found that out of 62 isolates of human origin, only one isolate (1.6%) was positive for the *mcr-1*. A higher incidence rate was reported by Rahman and Ahmed^19^ in India who found that out of 100 *A. baumannii* isolates of human origin, 20 (20%) were positive for *mcr-1* and Al-Kadmy et al.^37^ in Iraq found that out of 121 *A. baumannii* isolates of human and environmental origin, 89 (73.5%) isolates carried the *mcr-1* gene. However, in Iran, Khoshnood et al.^23^ reported that none of the 70 identified *A. baumannii* isolates carried the *mcr-1* resistance gene.

According to our knowledge, this study is the first to report the presence of the *mcr-1* gene in a human clinical *A. baumannii* isolate in Egypt. This finding raises issues to investigate other *mcr* genes in *A. baumannii* in future studies*.* This plasmid-mediated mechanism is important because it increases the horizontal transferability of colistin resistance of *A. baumannii* in clinical settings. Furthermore, colistin resistance may occur because of *pmrCAB* overexpression and/or mutations. Further studies are recommended to confirm the biological functions of point mutations of *pmr* genes and their role in colistin resistance. Strategies are required to stop the spread and evolution of such genes. Our research sheds light on the complexity of colistin resistance in *A. baumannii*. Further research is required to correlate colistin resistance and its molecular mechanisms to strain types and to understand other colistin resistance mechanisms such as studying the *lpx* genes. Moreover, it is vital to establish guidelines regarding the use of colistin to inhibit the emergence of resistance.

## Supplementary Information

Supplementary Information.

## Data Availability

*A. baumannii* strains are available from the authors. All data are fully available without restriction. All relevant data are within the manuscript and its Supporting Information files. The following are the GenBank accession numbers for nucleotide sequences: BankIt2586606 pmrA16 ON614677; BankIt2586606 pmrA41 ON614678; BankIt2586606 pmrA85 ON614679; BankIt2586606 pmrA71 ON614680; BankIt2586606 pmrA1 ON614681; BankIt2586606 pmrA44 ON614682; BankIt2586606 pmrA4 ON614683; BankIt2586606 pmrA43 ON614684; BankIt2586606 pmrB16 ON614685; BankIt2586606 pmrB41 ON614686; BankIt2586606 pmrB85 ON614687; BankIt2586606 pmrB71 ON614688; BankIt2586606 pmrB1 ON614689; BankIt2586606 pmrB44 ON614690; BankIt2586606 pmrB4 ON614691; BankIt2586606 pmrB43 ON614692; BankIt2586606 pmrC16 ON614693; BankIt2586606 pmrC41 ON614694; BankIt2586606 pmrC85 ON614695; BankIt2586606 pmrC71 ON614696; BankIt2586606 pmrC1 ON614697; BankIt2586606 pmrC44 ON614698; BankIt2586606 pmrC4 ON614699; BankIt2586606 pmrC43 ON614700.
